# Protection of Chloroplast Membranes by VIPP1 Rescues Aberrant Seedling Development in *Arabidopsis* *nyc1* Mutant

**DOI:** 10.3389/fpls.2016.00533

**Published:** 2016-04-28

**Authors:** Lingang Zhang, Makoto Kusaba, Ayumi Tanaka, Wataru Sakamoto

**Affiliations:** ^1^Institute of Plant Science and Resources, Okayama UniversityOkayama, Japan; ^2^Graduate School of Science, Hiroshima UniversityHiroshima, Japan; ^3^Institute of Low Temperature Science, Hokkaido UniversityHokkaido, Japan

**Keywords:** chlorophyll degradation, cotyledon development, NYC1, VIPP1, chloroplast membrane integrity, oxidative damage

## Abstract

Chlorophylls (Chl) in photosynthetic apparatuses, along with other macromolecules in chloroplasts, are known to undergo degradation during leaf senescence. Several enzymes involved in Chl degradation, by which detoxification of Chl is safely implemented, have been identified. Chl degradation also occurs during embryogenesis and seedling development. Some genes encoding Chl degradation enzymes such as Chl *b* reductase (CBR) function during these developmental stages. *Arabidopsis* mutants lacking CBR (NYC1 and NOL) have been reported to exhibit reduced seed storability, compromised germination, and cotyledon development. In this study, we examined aberrant cotyledon development and found that NYC1 is solely responsible for this phenotype. We inferred that oxidative damage of chloroplast membranes caused the aberrant cotyledon. To test the inference, we attempted to trans-complement *nyc1* mutant with overexpressing VIPP1 protein that is unrelated to Chl degradation but which supports chloroplast membrane integrity. VIPP1 expression actually complemented the aberrant cotyledon of *nyc1*, whereas stay-green phenotype during leaf senescence remained. The swollen chloroplasts observed in unfixed cotyledons of *nyc1*, which are characteristics of chloroplasts receiving envelope membrane damage, were recovered by overexpressing VIPP1. These results suggest that chloroplast membranes are a target for oxidative damage caused by the impairment in Chl degradation. Trans-complementation of *nyc1* with VIPP1 also suggests that VIPP1 is useful for protecting chloroplasts against oxidative stress.

## Introduction

At the onset of leaf senescence, an important physiological process at the final stage of leaf development, a leaf is reallocated from a sink to a source organ, from which macromolecules synthesized during the sink phase are degraded and are subsequently redistributed into the upper tissues or reproductive organs. Various factors such as transcriptional regulations, phytohormones, reactive oxygen species (ROS), and environmental factors influence leaf senescence progression (Lim et al., 2007; Woo et al., 2013). The major targets of the degradation processes have been demonstrated to reside in chloroplasts: macromolecules in chlo roplasts, such as protein, lipid, nucleic acid and pigment, undergo degradation (Krupinska, 2006; Hörtensteiner, 2013; Kato and Sakamoto, 2013; Sakamoto and Takami, 2014). Furthermore, autophagy has been demonstrated to incorporate chloroplasts during senescence (Ishida and Yoshimoto, 2008; Wada et al., 2009). These degradation processes contribute to the efficient recycling and redistribution of nitrogen compounds.

Among the degraded compounds in senescing leaves, chlorophyll (Chl) is the most abundant pigment. Higher plants contain both Chl *a* and Chl *b* in the light harvesting antennae and only Chl *a* in the reaction center of Photosystem I (PSI) and PSII. Chl is necessary for photosynthesis because the excitation energy received by Chl drives the water splitting reaction and subsequent electron transport. In contrast, an excited state of free Chl is toxic because it generates ROS ultimately leading to cell death. Chl degradation is therefore implicated as a safe means of detoxification (Tanaka and Tanaka, 2011; Hörtensteiner, 2013). Conversion of Chl *b* to Chl *a* mediated by Chl *b* reductase (CBR) is an initial step for Chl degradation (Ito et al., 1996; Rüdiger, 2002; Kusaba et al., 2007) because only Chl *a* undergoes the subsequent degradation pathway that involves (i) de-chelation of Chl *a* (pheophytin), (ii) removal of phyto-tail (pheophorbide *a*), and (iii) oxygenation of rings, leading to the production of non-toxic primary fluorescent catabolites. Mutations that impair any of the reactions above give rise to a ‘stay-green’ phenotype in which leaves implement senescence but retain Chl (Kusaba et al., 2013). This phenotype, designated as cosmetic stay-green, is apparent in many plant species such as *Arabidopsis* (Oh et al., 2004; Ren et al., 2007), rice (Kusaba et al., 2007; Park et al., 2007), pea (Armstead et al., 2007; Sato et al., 2007), tomato (Cheung et al., 1993; Akhtar et al., 1999), and soybean (Luquez and Guiamét, 2002; Fang et al., 2014).

Reportedly, Chl degradation takes place not only in senescing leaves but also during embryogenesis and cotyledon development. De-greening of developing embryos is usually observed in *Arabidopsis* and other species (Chao et al., 1995; Nakajima et al., 2012). Some enzymes for Chl degradation pathway appear to be up-regulated at these stages. Although the physiological implications of Chl degradation during embryogenesis and cotyledon development remain unclear, it is likely that Chl are catabolized properly during embryo maturation. Supporting the importance of Chl degradation in embryo, Nakajima et al. (2012) reported that the *Arabidopsis* mutant lacking CBR, Non-Yellow Coloring 1 (NYC1) and “NYC1-Like” (NOL1) caused the impairment of seed maturation and storability. This observation strongly implies that Chl accumulated in seeds are liable to cause oxidative damage to photosystems or peroxidation of chloroplast membranes, by which seeds do not show lethality but show reduced fitness.

In this study, we hypothesize that one target for the damage caused by impaired Chl degradation is chloroplast membranes. Previously, we demonstrated that VIPP1 plays a crucially important role in protecting photosynthetic membranes (Zhang et al., 2012; Zhang and Sakamoto, 2013, 2015). VIPP1 was originally identified as being abundant in envelopes (Li et al., 1994). Based on the seedling-lethal phenotype in *vipp1* mutant lacking vesicle formation, VIPP1 was suggested to play a role in thylakoid formation through vesicle fusion (Kroll et al., 2001; Westphal et al., 2001). However, its precise function remains elusive. VIPP1 forms an extremely large homo-complex (>2 MDa) that is associated with inner envelope and thylakoid membranes. The disassembly/reassembly of the VIPP1 complex, often designated as VIPP1 functional particle, takes place according to the membrane damage (Aseeva et al., 2004; Liu et al., 2005, 2007). It is particularly interesting that we recently demonstrated that overexpression of VIPP1 prevents chloroplast membranes from being damaged against heat shock and hypotonic stress. Therefore, it is possible to assume that VIPP1 overexpression improves seed defects in *Arabidopsis* *nyc1* mutant. Our results demonstrate that this is indeed the case, corroborating our hypothesis that the membrane integrity can be disturbed by Chl remaining in seeds. Our results also corroborate that VIPP1 functions in preventing chloroplast membranes from oxidative damage.

## Materials and Methods

### Material Preparation and Growth Conditions

*Arabidopsis thaliana* ecotype Columbia (Col) was used as the wild type in this study. The stay-green mutants, *nyc1*, *nol*, and *BCG*, were described previously. Briefly, both *nyc1* (corresponding to AT4G13250) and *nol* (corresponding to AT5G04900) are T-DNA insertion mutants that were identified by Horie et al. (2009). *BCG* refers to *Arabidopsis* transformant with a transgene containing BC domain of *CAO* fused with *GFP* at its C-terminus (Sakuraba et al., 2010). *VIPP1-GFP*/*nyc1, VIPP1-GFP*/*nol*, and *VIPP1-GFP*/*BCG* were generated, respectively, by crossing *VIPP1-GFP*/Col (Zhang et al., 2012) with *nyc1*, *nol* or *BCG*. Homozygous transformants were selected from the third generation. In the case of *VIPP1-GFP*/*nyc1* and *VIPP1-GFP*/*nol*, the corresponding T-DNA insertions at *nyc1* and *nol* loci were verified using PCR (primers 5′-TCAGTAGCACAGTCTTTCGCTC-3′ and 5′-GCGTTATATGCAGCAGAAGC-3′ for *nyc1*; primers 5′-TGTTGGTCTCCCATCTGAAC-3′ and 5′-GGCTACTTGGAGTGGTTTCA-3′ for *nol*). Expression of *VIPP1-GFP*/*BCG* was confirmed by Western blotting with antibody against VIPP1 and GFP. Seeds of these lines were surface-sterilized and were kept at 4°C. After 2 days, the seeds were sown onto 0.7% MS agar plates supplemented with 1.5% (w/v) sucrose. Seedlings were growing under light (low light of 35 μmol photon m^–2^ s^–1^ or normal light of 70 μmol photon m^–2^ s^–1^) with 12 h/12 h light/dark cycle at a constant temperature of 22°C. After a 2-weeks growth period on MS medium, the seedlings were transferred to soil.

### Darkness Treatment and Re-greening of Cotyledons

Sterilized seeds of Col, *nyc1*, and *VIPP1-GFP*/*nyc1* were kept at 4°C for 2 days; then they were sown on MS medium and kept in darkness by coverage with aluminum foil for 5 days at 22°C. Subsequently the foil was removed and seedlings on the MS plates were exposed to the light with intensity of 70 μmol photon m^–2^ s^–1^. The seedling phenotypes of different lines were recorded after 0, 1, 5, or 9-days re-greening. Darkness treatment was also used to induce the senescence of detached mature leaves of Col, *nyc1*, and *VIPP1-GFP*/*nyc1*. The protocol of dark-induced leaf senescence was fundamentally similar to that described by Sato et al. (2009). The mature leaves detached from 4-weeks-old plants were incubated at 22°C in darkness for 5 days.

### Chlorophyll Measurement

Chlorophyll (Chl) was extracted with 80% (v/v) acetone. Chl *a* and Chl *b* were determined, respectively, by absorbance at 663 and 645 nm with a spectrophotometer (Amersham Biosciences, Sweden). Quantification of Chl were calculated following the equations of Arnon’s (1949) method: total Chl (μg ml^–1^) = 20.2 (A_645_) + 8.02 (A_663_), Chl *a* (μg ml^–1^) = 12.7 (A_663_) – 2.69 (A_645_), Chl *b* (μg ml^–1^) = 22.9 (A_645_) – 4.68 (A_663_).

### Microscopy Observation

The cotyledons of 12-days-old seedlings of different lines were used for microscopic observation, as described in a previous report (Zhang et al., 2012). The chloroplasts/plastids in living leaf tissues were examined using a fluorescence microscope (DSU-BX51; Olympus Corp.) under a 100× objective lens with oil. For observation of *Arabidopsis* seeds, autofluorescence from the intact seeds was monitored directly under a microscope (DSU-BX51; Olympus Corp.) with a filter set (U-MWIB2). Corresponding bright-field photographs were recorded simultaneously.

### Protein Exaction, SDS-PAGE, and Western Blotting

For immunobloting, total proteins of 12-days-old seedlings were extracted through grinding with loading buffer (125 mM Tris-Cl, pH 6.8, 2% [w/v] SDS, 5% [v/v] glycerol, 5% [v/v] 2-mercaptoethanol, and 0.05% [w/v] bromophenol blue) directly. The extract was denatured continuously at 95°C for 5 min. Samples were loaded on SDS-PAGE in the equal fresh weight. One SDS-PAGE was stained with Coomassie Brilliant Blue R 250 as loading control. The other gel was electroblotted onto polyvinylidene difluoride membrane (Atto Corp.). The membrane was blocked with 5% (w/v) milk in PBST buffer (50 mM sodium phosphate buffer, pH 7.5, 155 mM NaCl, and 0.05% [v/v] Tween 20) for 1 h. After three washes with PBST buffer, the membranes were incubated with anti-VIPP1 (Aseeva et al., 2007) for 2 h. After washing three times again with PBST buffer, the membranes were incubated with second antibodies for 2 h. Proteins were detected using an ECL chemiluminescence detection system (Amersham Biosciences Corp.) as described in a previous report (Sakamoto et al., 2003).

### Histochemical Detection of H_2_O_2_

*In situ* detection of hydrogen peroxide was performed by staining with 3,3′-diaminobenzidine (DAB, Sigma-Aldrich) according to the previous method (Daudi et al., 2012). Briefly, the detached cotyledons of different lines were infiltrated for 5 min under gentle vacuum with 1 mg/mL DAB containing 0.05% (v/v) Tween 20 and 10 mM sodium phosphate buffer (pH 7.0). At least five independent plants were used as biological replicates, and three cotyledons were sampled from each plant. Staining reaction was terminated 5 h after DAB infiltration. The pigments in the DAB-stained seedlings were removed with ethanol: acetic acid: glycerol (3:1:1) in a water bath at 95°C for 15 min. DAB staining was observed under white light and photographed.

## Results

### Aberrant Seedling Development in *nyc1*

In the course of characterizing *Arabidopsis* stay-green mutants or transgenic lines that are impaired in Chl degradation, we found that some showed defective growth at the seedling stage. We particularly examined the mutants lacking NYC1 and NOL: the enzymes involved in the first step of Chl degradation by conversion of Chl *b* to Chl *a*. It has been demonstrated that NYC1 and NOL act redundantly or differentially in *Arabidopsis* because only *nyc1* mutant exhibits the stay-green leaf phenotype (Horie et al., 2009). In rice, in contrast, both NYC1 and NOL act synergistically by forming a heterocomplex in thylakoid membranes: a mutant lacking either NYC1 or NOL shows stay-green (Kusaba et al., 2007; Sato et al., 2009).

**FIGURE 1:**
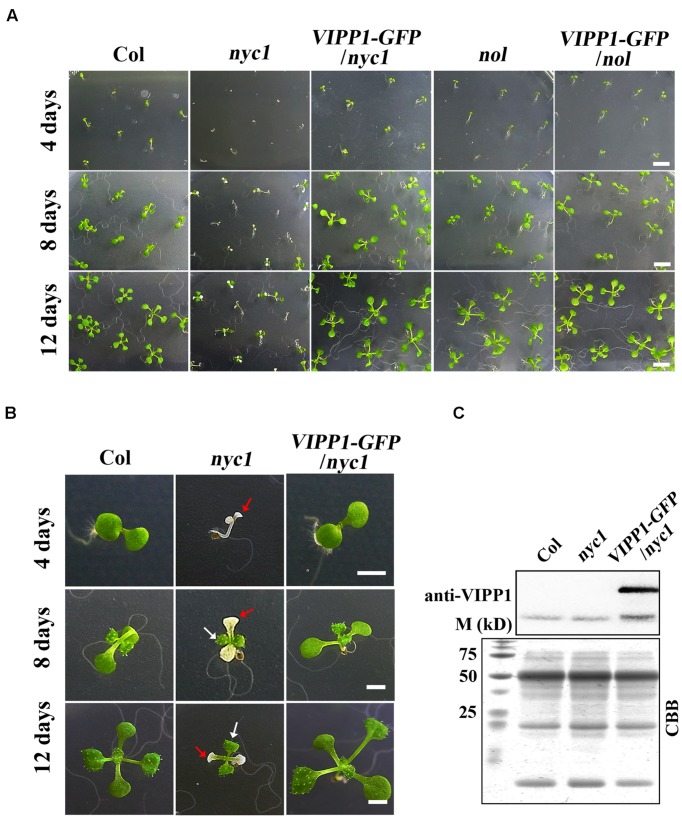
**Defective seedling growth in ***nyc1*** and its recovery in ***VIPP1-GFP/nyc1.*** (A)** Germination and seedling development of Col, *nyc1*, *VIPP1-GFP/nyc1*, *nol,* and *VIPP1-GFP/nol* on MS-agar plate supplemented with 1.5% sucrose under normal light with 12 h/12 h light/dark cycle at 22°C. Photographs of the seedlings were taken 4, 8, or 12 days after sowing, respectively, as shown at the left. Bars = 5 mm. **(B)** Close-up view of representative seedlings of Col, *nyc1,* and *VIPP1-GFP/nyc1* during seedling development at 4, 8, and 12 days after sowing. In *nyc1*, the cotyledon was defective and white (red arrows), whereas the first true leaf was green (white arrows). Bars = 2 mm. **(C)** Immunoblots of total protein extract from Col, *nyc1,* and *VIPP1-GFP/nyc1* (12-days-old individuals as shown in **(B)** were probed with anti-VIPP1. Loading was based on the equal total protein. Proteins stained with Coomassie Brilliant Blue (CBB) after SDS-PAGE are shown as loading control at the bottom. M denotes the molecular marker shown along with size.

When the seeds from *Arabidopsis* *nyc1* and *nol* were sown on MS agar plates supplemented with sucrose, we observed that *nyc1* developed chlorotic cotyledons (**Figures 1A,B**). In contrast, *nol* developed normal-appearing cotyledons. No defects in seedling were detected. Aberrant seedlings in *nyc1* were small and visible soon after cotyledon development (at least 4 days after sowing), showing small white or pale-green cotyledons occasionally leading to wilting. We have used at least three batches of seed stocks and reproducibly observed this aberrant seedling development only in *nyc1* (81% of seedling exhibited white or pale cotyledons). Despite the effect in cotyledons, first and subsequent true leaves emerged normally and were green (**Figure 1B**). When transplanted to soil and further grown for 2 weeks, *nyc1* seedlings appeared to grow normally, showing no visible phenotypes in our growth condition. At this stage, Chl *a*/*b* ratio of these plants grown in soil showed no marked difference between wild type Columbia and *nyc1*. Nakajima et al. (2012) reported that *nyc1nol* double mutant showed drastic reduction of germination rate along with prolonged storage of the seeds. That impaired germination was possibly induced by toxic Chl breakdown products. For this study, we sowed the 3-months-old seeds of *nyc1* and *nol* mutant on MS medium, which revealed that the germination rate of both mutants is similar with that of wild type (**Figure 1A**). These results suggest that *nyc1* has a defect in cotyledon formation, but it has no profound effect on the lateral leaf development.

### Cotyledon Re-greening of *nyc1* Was Affected by Light Intensity

**FIGURE 2:**
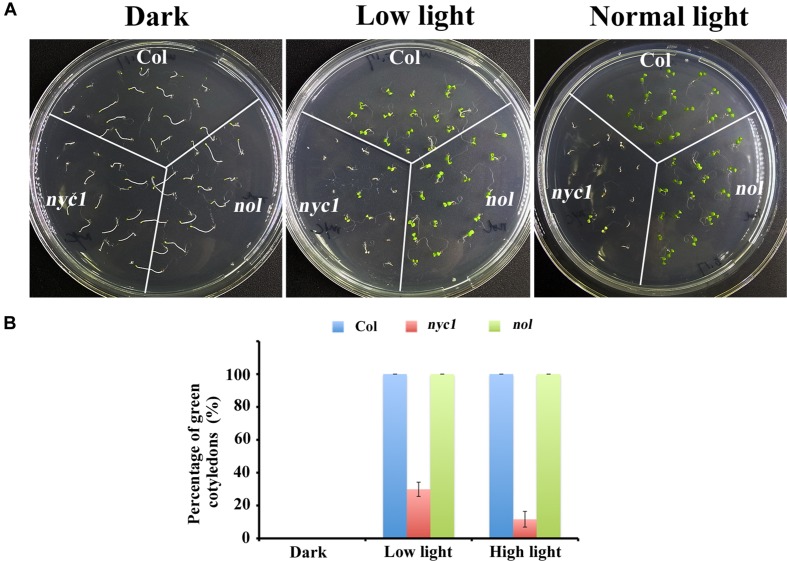
**Effect of light on germination and seedling development of Col, ***nyc1**,* and ***nol***. (A)** Photographs of 5-days-old seedlings from Col, *nyc1,* and *nol*. Sterilized seeds were sown on MS-agar plate and were kept under three light conditions: darkness (plate was covered with aluminum), low light (30 μmol photon m^–2^ s^–1^), and normal light (70 μmol photon m^–2^ s^–1^). **(B)** Rate of green cotyledons over all seedling in Col (Blue), *nyc1* (red), and *nol* (green) measured under three light conditions as noted at the bottom. Data shown in **(B)** are means of three independent experiments with error bars representing the SD.

Given that *nyc1/nol* seeds accumulate chlorophyll-derived compounds as detected by excess fluorescence in Nakajima et al. (2012), the aberrant seedling development is probably caused by oxidative stress and is therefore light-dependent. To test this possibility, we grew Col, *nyc1,* and *nol* under three light conditions (darkness, low light of 35 μmol photon m^–2^ s^–1^, and normal light of 70 μmol photon m^–2^ s^–1^) and observed germination and cotyledon greening. Again, no great difference in germination rates was found between wild type and two mutants, irrespective of light conditions (**Figures 2A,B**). In contrast, cotyledon re-greening of *nyc1* mutant differs with that of Col and *nol* under different light irradiation (**Figures 2A,B**). In *nyc1*, cotyledons presented various colors: green, pale-green, or white. The ratio of green cotyledons over germinated seedlings was 11.8%, which in turn increased to 28.7% when the light intensity was decreased to 35 from 70 μmol photon m^–2^ s^–1^. Therefore, re-greening of the *nyc1* cotyledon appeared to result from photo-oxidative damage.

### Expression of VIPP1-GFP Recovers Aberrant Seedling Growth in *nyc1*

In this study, we attempted to overexpress VIPP1 in *nyc1* to test if the aberrant cotyledon development is mitigated. We reported previously that VIPP1 which is C-terminally tagged with GFP (VIPP1-GFP) rescues seedling lethality of *vipp1* knock-down (*vipp1-kd*) and knock-out (*vipp1-ko*) mutants (Zhang et al., 2012). Moreover, the transgenic line overexpressing VIPP1-GFP (termed *VIPP1-GFP*/Col) were shown to increase tolerance to recovery from heat shock when detached mature leaves were examined. To this end, we crossed *VIPP1-GFP*/Col with *nyc1*. The resulting progeny were further characterized to obtain *VIPP1-GFP/nyc1* that harbored both *nyc1* allele and *VIPP1-GFP* transgene as homozygous. As expected, our Western blotting confirmed substantial accumulation of VIPP1-GFP in *VIPP1-GFP/nyc1* (**Figure 1C**), which appeared to be comparable to that described in a previous report (Zhang et al., 2012). Visual inspection of *VIPP1-GFP/nyc1* revealed that it grew normally at the post-germination stage, representing normal cotyledons with size equivalent to that of wild type Columbia (Col; **Figures 1A,B**). Moreover, chlorotic cotyledons were never detected in the progeny of *VIPP1-GFP/nyc1.* These results demonstrated that overexpression of VIPP1 can trans-complement the aberrant growth of *nyc1* at the seedling stage.

### VIPP1 Overexpression Promote the Greening of Yellow *nyc1* Cotyledon

**FIGURE 3:**
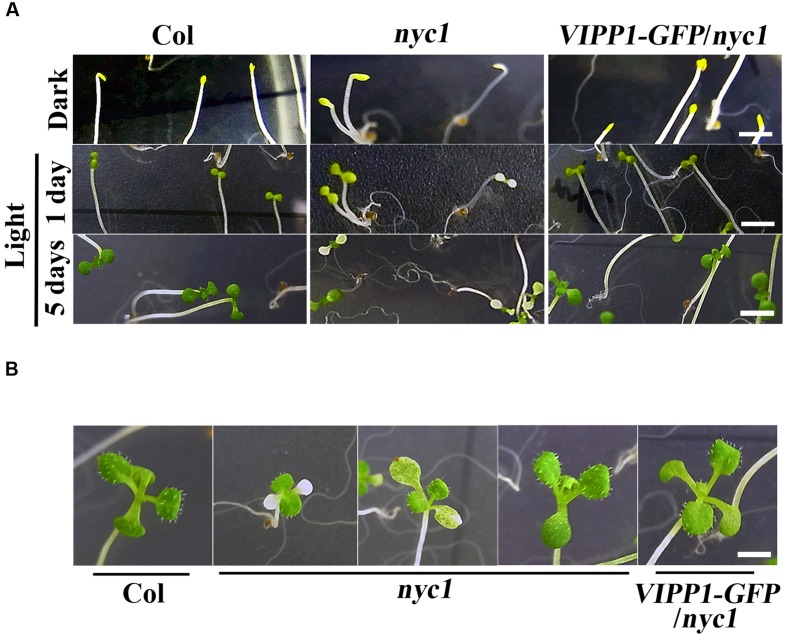
**Re-greening of yellow cotyledons of Col, ***nyc1**,* and ***VIPP1-GFP/nyc1***. (A)** Seedling phenotypes of Col, *nyc1,* and *VIPP1-GFP/nyc1*. Seeds of Col, *nyc1,* and *VIPP1-GFP/nyc1* were sown on MS-agar plate and grown further for 5 days in darkness (top panel) with subsequent exposure to normal light (70 μmol photon m^–2^ s^–1^) for 1 day (middle panel) or for 5 days (bottom panel). Bars = 5 mm. **(B)** Photographs of seedlings from Col, *nyc1,* and *VIPP1-GFP/nyc1*. These were taken 9 days after 5-days growth in darkness. Bars = 5 mm.

To address the protection effect of VIPP1 on *nyc1* cotyledons further, we next examined re-greening processes during transition from etioplast to chloroplast. Seeds of Col, *nyc1,* and *VIPP1-GFP/nyc1* were germinated on MS-agar plates. Prolonged incubation in darkness for 5 days led these seedlings to develop long hypocotyl and etiolated cotyledon (**Figure 3A**, top panel). Subsequently, these etiolated seedlings were exposed to normal light (70 μmol proton m^–2^ s^–1^). Cotyledons of Col and *VIPP1-GFP/nyc1* turned green from yellow (**Figure 3A**), implying that etioplasts of these yellow cotyledons were properly converted into chloroplasts upon light irradiation. However, cotyledons of *nyc1* exhibited a range of colors from white to green (**Figure 3A**), indicating that some etioplasts cannot form chloroplasts. Even after prolonged light exposure of up to 12 days, some cotyledons of *nyc1* mutant did not turn green, but true leaves developed normally (**Figure 3B**). We conclude that the aberrant cotyledon in *nyc1* is caused partly by photo-oxidative damage in chloroplast membranes, which impairs the etioplast–chloroplast transition.

### *VIPP1-GFP/nyc1* Is Stay-Green Similarly to *nyc1*

**FIGURE 4:**
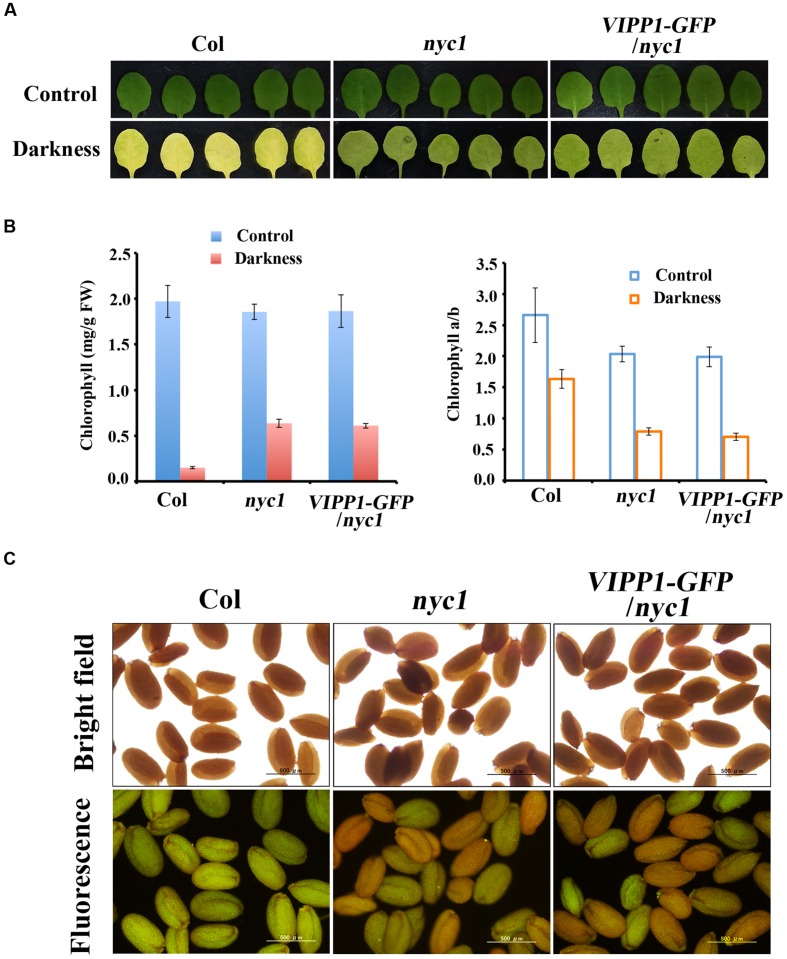
**Stay-green phenotype and retention of Chl and fluorescence in ***nyc1*** and ***VIPP1-GFP/nyc1***. (A)** Dark-induced senescence of mature leaves from Col, *nyc1,* and *VIPP1-GFP/nyc1*. Detached leaves of 3-weeks-old plants were incubated in darkness for 5 days at 22°C. Photographs of representative leaves before and after dark treatment are shown, respectively, as control (top panel) and darkness (bottom panel). **(B)** Total Chl content (left) and Chl *a/b* ratio (right) were calculated from the leaves as shown in **(A)**. Three independent experiments were performed and error bars (SD) are represented as SD. Values before and after dark treatment are shown, respectively, as control (blue) and darkness (red). **(C)** Fluorescence emission of seeds from Col, *nyc1,* and *VIPP1-GFP/nyc1* detected by microscopy (bottom panel). Photographs of the same seeds taken by bright field are shown at the top.

A possibility that explains trans-complementation of *nyc1* with VIPP1-GFP is that VIPP1 assists in degrading chlorophylls for unknown reasons, although it is unlikely. This possibility can be excluded by observing the stay-green phenotype of *nyc1* and *VIPP1-GFP/nyc1*: these plants are expected to contain higher levels of Chl during leaf senescence. To examine this conjecture, we followed a previous report (Sato et al., 2009) and adopted dark-induced senescence of detached mature leaves. After incubation in darkness for 5 days, leaves of Col turned yellow and Chl were degraded, whereas *VIPP1-GFP/nyc1* leaves, as well as those of *nyc1*, remained green because of Chl retention (**Figures 4A,B**). Moreover, reduced Chl *a/b* ratios in *nyc1* and *VIPP1-GFP/nyc1* indicated that both were deficient in CBR activity and that they indeed accumulated more Chl *b* (**Figure 4B**, right panel). In an earlier study, retention of Chl in *nyc1* was likely to vary among the seeds, some exhibiting high fluorescence and others showing lower fluorescence (Nakajima et al., 2012). Although the exact compounds emitting fluorescence remained unclear, high fluorescence was regarded as derived from Chl degradation products. Similarly to *nyc1*, seeds of *VIPP1-GFP/nyc1* also showed varied degrees of fluorescence (**Figure 4C**). Most of the wild-type seeds exhibited low fluorescence, indicating that a low level of Chl was retained in the wild-type seeds. These results demonstrate that overexpression of VIPP1 did not affect Chl degradation of the *nyc1* mutant.

### Envelope Damage Assessed by Chloroplast Swelling in *nyc1* Cotyledons

**FIGURE 5:**
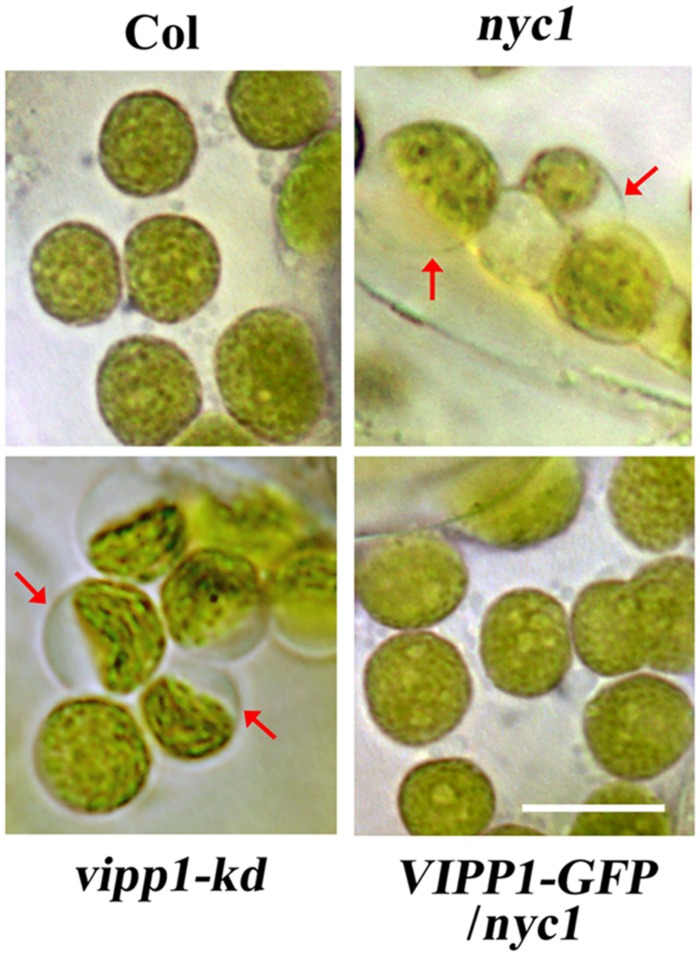
**Chloroplasts of unfixed cotyledons from ***nyc1*** and ***BCG*** show balloon-like structure characteristic to defective envelope.** Representative photographs of chloroplasts in unfixed cotyledons from Col, *nyc1*, *vipp1-kd*, and *VIPP1-GFP/nyc1* were taken by microscopic observation under bright field. Balloon-like chloroplasts that are characteristic of weakened envelope integrity (red arrows) were detected only in *vipp1-kd* and *nyc1* but recovered in the lines overexpressing VIPP1-GFP. Bars = 10 μm.

As confirmed by previously described data, trans-complementation with VIPP1 strongly suggests that the defective re-greening of *nyc1* is correlated with membrane damage in chloroplasts. Therefore, membrane integrity in chloroplasts, particularly that of envelopes, was assessed by observing chloroplasts in cotyledons. Our previous work demonstrated that the lack or decreased level of VIPP1 in *vipp1-ko* and *vipp1-kd* causes chloroplasts to form swelling of envelopes when chloroplasts in leaf tissues are observed directly: an enlarged stroma area within the chloroplast engenders ‘balloon-like’ chloroplasts, which are only slightly detectable in wild type (**Figure 5**). It is interesting that we were able to observe chloroplasts/plastids in white cotyledons of *nyc1* that showed the swelling phenotype under microscopy (see Materials and Methods). Although, the appearance of such chloroplasts in these lines was rare (approximately 1% or less), it was detected consistently. However, we never detected them in Col. More importantly, the appearance of balloon-like chloroplasts in *nyc1* were recovered by expressing *VIPP1-GFP* (**Figure 5**). These results strongly support our supposition that the recovery of the aberrant cotyledon development results from the improved tolerance of chloroplast membranes against oxidative damage.

## Discussion

The aberrant cotyledon phenotype described in this study (**Figure 1**) has been briefly reported by Nakajima et al. (2012), in which the authors rather focused on the effect of impaired Chl degradation on seed storability. They noted that *nyc1/nol* double mutant displays the aberrant cotyledon that looked similar to what is reported in this study, showing plant-to-plant variability from white to pale yellow colors. However, this phenotype has not been investigated further. Here, we demonstrated that it is *nyc1*, but not *nol*, from which the aberrant phenotype derives: it is also the case in which in *Arabidopsis*, *nyc1* shows a cosmetic stay-green phenotype during leaf senescence, whereas *nol* does not (Horie et al., 2009). These findings together suggest that unlike rice, NYC1 plays a major role in Chl degradation not only of senescing leaves but also of seed maturation. Furthermore, we demonstrate that light intensity affects the appearance of aberrant cotyledons (**Figure 2**), which partly explains why the penetrance of the cotyledon color phenotype varied. Chl acts as a potential photosensitizer when not integrated into the photosystems (Hörtensteiner, 2004). It is therefore likely that Chl degradation in seed tissues must be tightly regulated to maximize fitness. Although, no difference in germination rate was detected in our study (**Figure 2**), retention of Chl in the mature seeds has been implicated as correlated with the lower seed germination rate (Jalink et al., 1998). We detected only the aberrant cotyledon, not lowered germination rate, probably because we used seeds that had been stored only for a short period (3 months).

**FIGURE 6:**
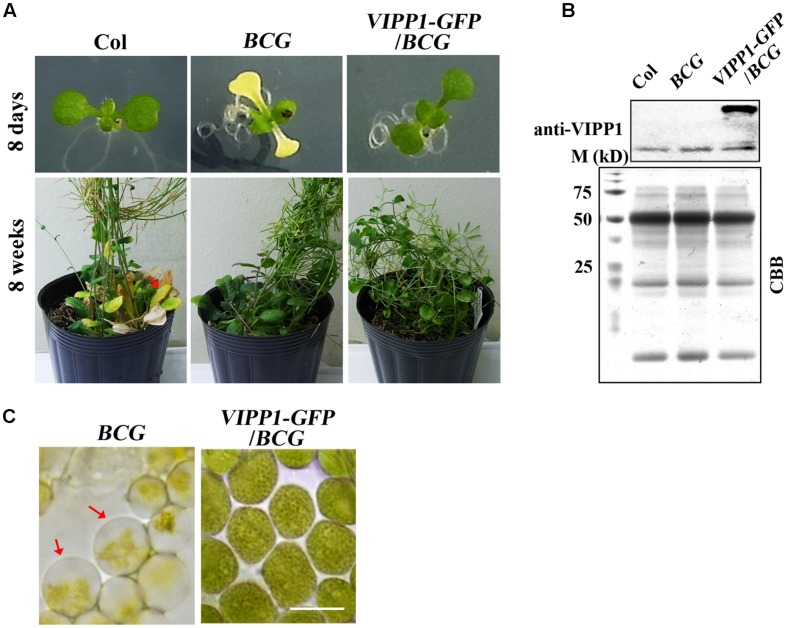
**Defective seedling growth in ***BCG*** and its recovery in ***VIPP1-GFP/BCG***. (A)** Photographs of representative seedlings (8-days-old, top panel) and mature plants (8-weeks-old, bottom panel) of Col, *BCG,* and *VIPP1-GFP/BCG*. Aberrant cotyledon development of *BCG* shown in yellow is rescued by VIPP1 overexpression in *VIPP1-GFP/BCG* (top panel), whereas late senescence phenotype is retained in both lines (bottom panel). **(B)** Immunoblot of total protein extract from Col, *BCG,* and *VIPP1-GFP/BCG* was probed with anti-VIPP1 as presented in **Figure 1C**. **(C)** Recovery of the balloon-like chloroplasts in *BCG* (indicated by red arrows) by expressing *VIPP1-GFP.* Bars = 10 μm.

We reasoned that our result of trans-complementation should be confirmed by some other means because we used only one allele of *nyc1*. A stay-green transgenic line (termed *BCG*) showing the phenotype similar to *nyc1* was reported by Sakuraba et al. (2012). Instead of knockout in *NYC1*, *BCG* overproduces Chl *b* oxygenase (CAO) that engenders increased Chl *b* levels. As a consequence, *BCG* was shown to exhibit a prolonged stay-green in senescing leaves. CAO consists of three domains (respectively designated as A, B, and C), and A domain negatively regulates CAO accumulation *in vivo*. To circumvent this negative regulation, *BCG* expressed CAO that contains only B and C domains: because BC-CAO is tagged with GFP, the resulting transgene (and transgenic line) was designated as *BCG* (Sakuraba et al., 2010). We found that under our growth condition (70 μmol photon m^–2^ s^–1^), *BCG* cotyledons show white or pale yellow as seen in *nyc1* (**Figure 6**). Interestingly, introduction of *VIPP1-GFP* into *BCG* recovers the aberrant cotyledon phenotype (**Figure 6**). *VIPP1-GFP/BCG* apparently retained the stay-green phenotype in senescing leaves. Moreover, the envelope damage assessed by the balloon-like chloroplasts in cotyledons was also recovered by *VIPP1-GFP* (**Figure 6**). Results from *VIPP1-GFP/BCG* were consistent with *VIPP1-GFP/nyc1*, leading us to confirm that VIPP1 trans-complemented aberrant cotyledon development because of the disturbance in Chl degradation.

The similar phenotype between *nyc1* and *BCG* implies tight regulation of Chl levels during cotyledon development. In contrast to the cotyledon, however, true leaves of *nyc1* and *BCG* develop normally because of their distinct differentiation paths in these two organs. Chloroplasts in true leaves originate from proplastids of apical meristem cells, where robust thylakoid membranes are formed in a light-dependent manner. Chloroplasts in cotyledons are differentiated upon germination from pre-existing plastids, which contain thylakoid membranes developed during embryogenesis. Together, our data enable us to conclude that Chl degradation is necessary for the initial stage of plant life: seedling development.

Given the light-dependent chlorotic phenotype, we inferred that overproduction of Chl (and perhaps its degradation compounds detected as high fluorescence, **Figure 4**) tend to accumulate excitation energy that is not transferred to photosystems and ultimately engender production of toxic molecules such as ROS. Under this scenario, it was speculated that ROS accumulates more in *nyc1* than Col cotyledons. However, histochemical detection of hydrogen peroxide showed no significant difference (**Supplementary Figure S1**). Perhaps subtle and transient difference in ROS accumulation is sufficient to result in aberrant cotyledons. Sakuraba et al. (2010) has shown that in *BGC* plants, true leaves accumulate high ROS in a light-dependent manner, which suggests that excess Chl may lead to photo-oxidative stress. Reportedly, oxidative stress is one of the factors that affect the seed storability (Sattler et al., 2004). Another remaining question is whether excess Chl can associate with envelopes or in its vicinity, which subsequently affects envelope membrane integrity. We infer that it is unlikely, because Nakajima et al. (2012) reports that not only Chl but also LHCII exist in *nyc1* cotyledons. Although the physiological status of plastids in aberrant cotyledons is unclear, excess Chl appeared to form LHC, similarly to those in senescing leaves. A possibility that VIPP1 scavenges ROS directly is also unlikely: no such implication has been suggested.

Numerous mutants showing white cotyledons have been reported in *Arabidopsis* (e.g., *sco1*, *cyo1*, *sco2*, *sig2*). For example, *cyo1* exhibits white cotyledons; CYO1 has been shown to have disulfide reductase activity (Shimada et al., 2007). Preferential expression of CYO1 in young seedlings implicates that, although the precise substrate of CYO1 remains unclear, redox regulation is crucial in cotyledons. Unlike *nyc1*, however, the appearance of white cotyledons in *cyo1* is independent of the light intensity and does not result from photobleaching. It has been suggested that ROS generation in chloroplasts renders membranes sensitive to oxidative stress damage (Mahajan and Tuteja, 2005). Chloroplast membrane stability has been compromised when membrane lipid peroxidation has resulted from ROS (Anjum et al., 2011; Lata et al., 2011). Therefore, it seems reasonable to assume that one target damaged by Chl over-accumulation in the cotyledons of *nyc1* is the chloroplast membrane.

Supporting this assumption, results of this study show that chloroplast envelopes in green cotyledons of *nyc1* sustained membrane damage, as evidenced by balloon-like chloroplasts (**Figure 5**). Direct observation of chloroplasts in unfixed leaf tissues enables us to infer that chloroplast swelling is indicative of hypotonic emphasize that disturbs envelope membrane potential. Originally, we used this method to ascertain the important role of VIPP1 in envelope integrity: *vipp1-kd* shows the typical balloon-like chloroplasts (Zhang et al., 2012). VIPP1 is found specifically in photosynthetic organisms and is a multifunctional protein originated from bacterial ancestral protein PspA. It has a C-terminal extension that does not exist in PspA, has no transmembrane helix, and associates with membranes by forming large complexes (Zhang and Sakamoto, 2015). The large homo complex consists of VIPP1 function particles that mutually associate, thereby forming various dot-like, rod-like, and lattice-like structures. Various functions such as vesicle formation, maintenance of photosystems, protein import across thylakoid membranes, envelope maintenance, and membrane fusion have been inferred. Along with the lethal phenotype of *vipp1-ko*, there observations suggest that VIPP1 plays a fundamental role in maintaining chloroplast membranes. Trans-complementation of *nyc1* with VIPP1 overexpression also suggests an important role of VIPP1 in chloroplast maintenance.

## Conclusion

We drew two conclusions from our current study. First, one target for photo-oxidative damage caused by impaired Chl degradation is chloroplast membranes, particularly during seedling formation. VIPP1 apparently has little effect on stay-green phenotype in *nyc1* because membranes undergo extensive destruction during leaf senescence. In addition, the possibility that VIPP1 assists in degrading Chl is excluded because none of *VIPP1-GFP/nyc1* or *VIPP1-GFP/BCG* recovered stay-green during leaf senescence. A second conclusion is that VIPP1 can prevent chloroplasts from receiving damage against oxidative stress. In this regard, trans-complementation of *nyc1* by overexpressing VIPP1 is related to tolerance against heat shock in *VIPP1-GFP/*Col, as found from our earlier study. The present study therefore reinforces our notion that VIPP1 is important to protect chloroplast membranes against environmental stresses.

Stay-green is a key plant trait with wide usage for managing crop production. Functional stay-green can increase biomass and grain yield of crop through delayed senescence of the photosynthetic organs (Kusaba et al., 2013). Although cosmetic stay-green is unlikely to contribute to high yields, it is useful for high-quality food production (e.g., evergreen leaves, grasses). The shortcoming of Chl retention inside seeds appears to accompany a low germination rate (Parcy et al., 1997; Nakajima et al., 2012), which can be expected to hinder the practical application of stay-green trait. In this paper, we presented the possibility that VIPP1 overcomes such weakness when its overexpression is combined with stay-green.

## Author Contributions

WS, MK, AT designed the work; LZ, WS, AT prepared the materials; LZ and WS performed the experiments; LZ, WS, MK, AT analyzed the data; WS and LZ wrote the manuscript.

## Conflict of Interest Statement

The authors declare that the research was conducted in the absence of any commercial or financial relationships that could be construed as a potential conflict of interest.

## Supplementary Material

FIGURE S1 **Histochemical detection of H_2_O_2_ in cotyledons.** Seedlings from Col, *nyc1*, and *VIPP1-GFP/nyc1* were grown on MS plates for 2 weeks under light with intensity of 70 μmol photons m^–2^ s^–1^. Detached cotyledons of these lines were used for H_2_O_2_ detection with DAB staining as described in Materials and Methods. Bars = 0.5 mm.
