# Supplementary figures and images for: Protection of Chloroplast Membranes by VIPP1 Rescues Aberrant Seedling Development in Arabidopsis nyc1 Mutant
Source: Front Plant Sci. 2016 Apr 28;7:533. doi: 10.3389/fpls.2016.00533 (PMC4848304; doi:10.3389/fpls.2016.00533)

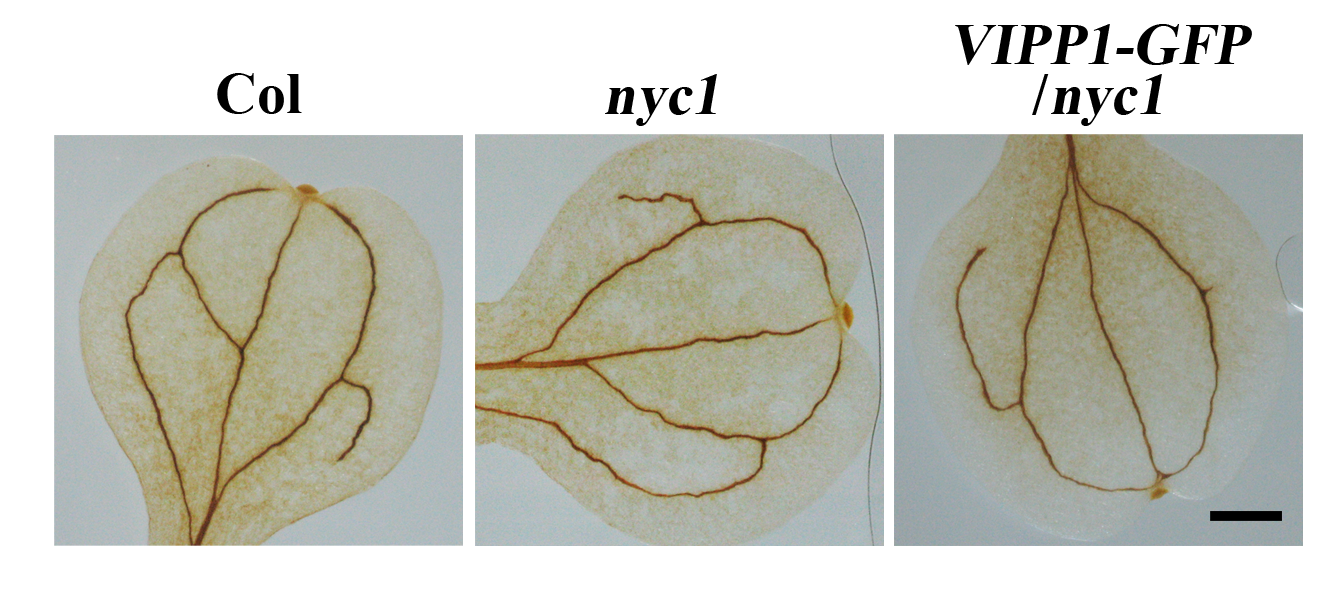

Supplement: FIGURE S1 — Histochemical detection of H2O2 in cotyledons. Seedlings from Col, nyc1, and VIPP1-GFP/nyc1 were grown on MS plates for 2 weeks under light with intensity of 70 μmol photons m–2 s–1. Detached cotyledons of these lines were used for H2O2 detection with DAB staining as described in Materials and Methods. Bars = 0.5 mm. [file Image_1.TIF]
